# The effect of 50% oxygen on PtCO_2_ in patients with stable COPD, bronchiectasis, and neuromuscular disease or kyphoscoliosis: randomised cross-over trials

**DOI:** 10.1186/s12890-020-1132-z

**Published:** 2020-05-07

**Authors:** Janine Pilcher, Darmiga Thayabaran, Stefan Ebmeier, Mathew Williams, Geraldine Back, Hamish Collie, Michael Richards, Susan Bibby, Ruth Semprini, Mark Weatherall, Richard Beasley

**Affiliations:** 1 Medical Research Institute of New Zealand, Private Bag 7902, Wellington, 6242 New Zealand; 2 Capital & Coast District Health Board, Wellington, New Zealand; 3 Victoria University of Wellington, Wellington, New Zealand; 4 University of Otago Wellington, Wellington, New Zealand

**Keywords:** Bronchiectasis, Carbon dioxide, COPD, Neuromuscular diseases, Oxygen

## Abstract

**Background:**

High-concentration oxygen therapy causes increased arterial partial pressure of carbon dioxide (PaCO_2_) in patients with COPD, asthma, pneumonia, obesity and acute lung injury. The objective of these studies was to investigate whether this physiological response to oxygen therapy occurs in stable patients with neuromuscular disease or kyphoscoliosis, and bronchiectasis.

**Methods:**

Three randomised cross-over trials recruited stable patients with neuromuscular disease or kyphoscoliosis (*n* = 20), bronchiectasis (*n* = 24), and COPD (n = 24). Participants were randomised to receive 50% oxygen and 21% oxygen (air), each for 30 min, in randomly assigned order. The primary outcome was transcutaneous partial pressure of carbon dioxide (PtCO_2_) at 30 min. The primary analysis was a mixed linear model.

**Results:**

Sixty six of the 68 participants had baseline PtCO_2_ values < 45 mmHg. The intervention baseline adjusted PtCO_2_ difference (95% CI) between oxygen and room air after 30 min was 0.2 mmHg (− 0.4 to 0.9), *P* = 0.40; 0.5 mmHg (− 0.2 to 1.2), *P* = 0.18; and 1.3 mmHg (0.7 to 1.8), *P* < 0.001, in the neuromuscular/kyphoscoliosis, bronchiectasis and COPD participants respectively.

**Conclusions:**

The small increase in PtCO_2_ in the stable COPD patients with high-concentration oxygen therapy contrasts with the marked increases in PaCO_2_ seen in the setting of acute exacerbations of COPD. This suggests that the model of studying the effects of high-concentration oxygen therapy in patients with stable respiratory disease is not generalisable to the use of oxygen therapy in the acute clinical setting. Appropriate studies of high-concentration compared to titrated oxygen in acute clinical settings are needed to determine if there is a risk of oxygen-induced hypercapnia in patients with neuromuscular disease, kyphoscoliosis or bronchiectasis.

**Trial registration:**

Australian New Zealand Clinical Trials Registry ACTRN12615000970549 Registered 16/9/15, ACTRN12615000971538 Registered 16/9/15 and ACTRN12615001056583 Registered 7/10/15.

## Background

Oxygen has the potential to elevate arterial partial pressure of carbon dioxide (PaCO_2_) in patients with chronic obstructive pulmonary disease (COPD) [1–5]. The incidence and magnitude is variable and it can occur in both stable and acute exacerbations of COPD [1, 5]. Oxygen-induced hypercapnia is likely to be clinically important in the setting of acute exacerbations of COPD. A randomised-controlled trial (RCT) comparing high-concentration and titrated oxygen (to achieve arterial oxygen saturation (SpO_2_) of 88 to 92%) in patients with an acute COPD exacerbation identified an over two-fold increase in mortality with high-concentration oxygen [6]. Consequently oxygen therapy guidelines recommend titration of oxygen therapy within this SpO_2_ range in patients with acute exacerbations of COPD, to avoid the risks of both hypoxaemia and hyperoxaemia [2, 3].

A number of mechanisms have been proposed to explain how oxygen therapy increases PaCO_2_. These include a reduction in hypoxic drive to breathe leading to decreased ventilation, and the release of hypoxic pulmonary vasoconstriction leading to worsening of ventilation-perfusion mismatch and increased alveolar deadspace [5, 7]. RCTs report high-concentration oxygen therapy increases PaCO_2_ in patients with asthma [8], pneumonia [9], and obesity [10–12]. This suggests oxygen-induced hypercapnia might occur in a range of respiratory conditions with abnormal gas exchange and/or reduced ventilation with respiratory failure.

Neuromuscular disease and kyphoscoliosis can lead to hypoventilation and chronic respiratory failure; airflow obstruction and ventilation-perfusion mismatch are both features of bronchiectasis. As acute respiratory illnesses complicate both of these conditions and can result in hypoxia and the need for oxygen therapy, it is important to establish whether these patients are at risk of oxygen-induced hypercapnia. Two small sleep studies with a total of 17 participants [13, 14] and one exercise study in 22 participants [15] have been performed in patients with cystic fibrosis, which demonstrated average transcutaneous partial pressure of carbon dioxide (PtCO_2_) increases between 4 [15] and 7.5 mmHg [13] during oxygen therapy compared to room air. In patients with neuromuscular disease data are limited to retrospective case examples [16] and a retrospective case series in eight patients with a range of neuromuscular diseases [17]. In the case series, low-flow oxygen (0.5-2 L/min) was associated with an elevation in PaCO_2_ by an average 28 mmHg, however the measurements were made up to six days after oxygen therapy.

The purpose of our randomised cross-over trials was to investigate the effects of 50% oxygen compared to 21% oxygen in patients with stable neuromuscular disease or kyphoscoliosis and patients with stable bronchiectasis. To assess the applicability of the results to the clinical setting, we also studied stable COPD patients, matched by severity of airflow obstruction to the bronchiectasis patients. Our hypothesis was that oxygen therapy would increase PtCO_2_ in all three trials.

## Methods

### Overview

This series of three double-blind cross-over trials randomised patients to the order they received 50% oxygen (“oxygen” intervention) and medical grade air containing 21% oxygen (“air” intervention). The trials recruited 20 patients with neuromuscular disease or kyphoscoliosis (Neuromuscular/kyphoscoliosis Study), 24 patients with bronchiectasis (Bronchiectasis Study) and 24 patients with COPD (COPD Study). Each trial was prospectively registered on ANZCTR (ACTRN12615000970549, ACTRN12615000971538 and ACTRN12615001056583, respectively), and had Health and Disability Ethics Committee approval (see Online Supplement). Inclusion and exclusion criteria are presented in Table 1, including the criteria by which Bronchiectasis and COPD participants were matched by airflow obstruction severity.

**Table 1:** Inclusion/Exclusion criteria

Study	Neuromuscular disease/ Kyphoscoliosis	Bronchiectasis	COPD
**Inclusion criteria**			
	Neuromuscular disease with ≥10% drop VC from sitting to lying or SNIP < 95% limit^**a**^*and/or* Kyphoscoliosis with an FVC < 65% predicted [18]^**b**^	Bronchiectasis as diagnosed by a doctor and confirmed with CT scan *and/or* Cystic fibrosis as diagnosed by a doctor	COPD, as diagnosed by a doctor
**Exclusion criteria**^**c**^			
Study Baseline PtCO_2_	≥60 mmHg	≥60 mmHg	≥60 mmHg
Age	< 14 years old	< 14 years old	< 16 years old
Comorbidities	COPD Morbid obesity^**d**^	COPD Morbid obesity^**d**^	Bronchiectasis Morbid obesity^**d**^
Spirometry	FEV_1_:FVC ratio ≤ 0.7, if participant is able to complete forced spirometry		FEV_1_:FVC ratio > 0.7 Inability to match FEV_1_ percentage predicted with a Bronchiectasis study participant^**e**^
Other		Infection with Burkholderia > 10 pack year smoking history	

*COPD* Chronic Obstructive Pulmonary Disease, *CT* computerised tomography, *FEV*_*1*_ Forced expiratory volume in 1 s, *FVC* Forced vital capacity, *PtCO*_*2*_ Transcutaneous partial pressure of carbon dioxide, *SNIP* Sniff nasal inspiratory pressure, *VC* Vital capacity (slow)

^**a**^For examples of neuromuscular disease diagnoses see Benditt & Boitano, 2013 [19]. The SNIP limit is a value under which 95% of healthy subjects are as based on work by Uldry & Fitting [20].

^**b**^Note that this may be calculated using arm span as per European Respiratory Society guidelines [21].

^**c**^All studies also excluded participants for any other condition which, at the investigator’s discretion, was believed may present a safety risk or impact the feasibility of the study or the study results

^**d**^Body mass index (BMI) ≥ 40 kg/m^2^

^**e**^To be a match, the COPD participant must have an FEV_1_ percentage predicted within an absolute value of 5% of the FEV_1_ percentage predicted for the bronchiectasis study participant (values inclusive). An exception to this was for the three bronchiectasis participants that had FEV_1_ percentage predicted values of 109% or higher, who were matched with COPD participants with an FEV_1_ percent predicted of 80% or over (i.e. participants in the mildest COPD severity category based on FEV_1_) [22]. The exception was made as it was not feasible to recruit matching COPD participants with FEV_1_ values within 5% and the required obstruction (FEV_1_/FVC < 0.7), as this would require them to have an FVC well in excess of their predicted value

Potentially eligible patients were recruited through Hutt Valley Hospital, Wellington Regional Hospital and the Medical Research Institute of New Zealand (MRINZ) patient lists, as well as newsletters and posters. Participants attended a single study visit at the MRINZ. After confirming eligibility, study baseline PtCO_2_, heart rate, and SpO_2_ were recorded via a SenTec transcutaneous monitor (SenTec AG, Switzerland), and respiratory rate by investigator observation.

Participants were then fitted with a full-face positive airway pressure mask (Respironics), attached to a Douglas Bag (Hans Rudolph) via CO_2_SMO adapter (Novametrix Medical Systems), one-way T valve, respiratory filter (Microgard II, Carefusion), tubing and three-way tap, all connected in series. Participants breathed room air for at least 5 mins to adjust to breathing through the equipment, and then breathed the intervention gas for 30 min. After each intervention, the mask was removed and there was a 30 min observation period breathing room air.

### Endpoint measurement

The primary endpoint was PtCO_2_ after 30 min.

Heart rate and PtCO_2_ were measured via SenTec. Respiratory rate, end tidal carbon dioxide (ETCO_2_), minute ventilation and dead space to tidal volume (VD/VT) were measured via CO_2_SMO (Model 8100), from which the tidal volume, volume of dead space, alveolar volume and alveolar minute ventilation were calculated (see Online Supplement for further detail regarding equipment methodology). Measurements were taken at T = 0 (following mask stabilisation and immediately prior to intervention), and at 10 min intervals during the intervention and observation periods. SpO_2_ on the SenTec display was covered during the intervention and washout periods to maintain investigator blinding. PtCO_2_ was monitored continuously; the intervention was stopped if values rose by ≥10 mmHg from T = 0.

The study statistician, who was not involved in study recruitment or visits, created computerised 1:1 randomisation sequences for each study. Randomisation codes were placed in sealed opaque envelopes and opened by the unblinded investigator following T = 0 measurements. The blinded investigator recorded all measurements from this point onwards.

### Analyses

The primary analysis was a mixed linear model with fixed effects for the T = 0 measurements, intervention, and randomisation order, and a random effect for participants to take into account the cross-over design. For all measures an interaction term was tested first to see if there was any difference between the interventions that depended on the time of measurement. As secondary analyses for all outcomes, the differences between interventions at each measurement time were analysed by similarly structured models, with addition of the fixed effect of the time of measurement and a random effect for each participant using a spatial exponential in time repeated measures variance-covariance matrix to account for the cross-over design. The results of this model for PtCO_2_ were compared between the COPD and Bronchiectasis study participants (as fixed effects), and were also adjusted for forced expiratory volume in one second (FEV_1_) percentage predicted. Finally the difference in proportions of participants with a change in PtCO_2_ of ≥ 4 mmHg and ≥ 10 mmHg from T = 0 were also estimated, as physiologically and clinically significant differences, respectively [8, 9, 23]. All estimates of differences are shown as oxygen minus room air.

### Software used

SAS version 9.4 was used.

### Sample size

The intended sample size for each cross-over study was 24 based on 80% power and a type I error rate of 5%, to detect a difference of 2.4 mmHg. This is half the difference found in a study of participants with obesity hypoventilation syndrome which reported a mean (SD) paired difference of 5 (4) mmHg [10].

## Results

### Participants

**Fig. 1:**
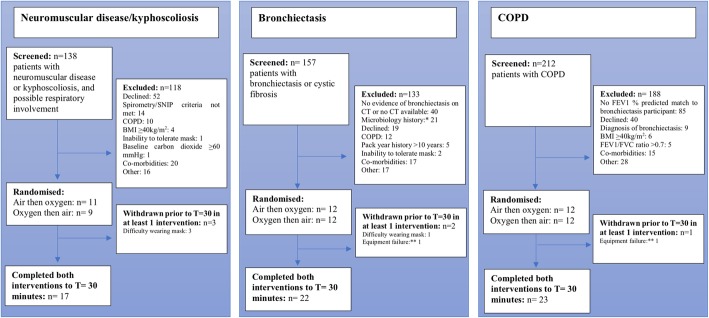
Flow of participants through studies. BMI: Body mass index, COPD: Chronic obstructive pulmonary disease, CT: Computerised tomography, FEV_1_: Forced expiratory volume in 1 s, FVC: Forced vital capacity, SNIP: Sniff nasal inspiratory pressure. *Burkholderia or other result at the investigator’s discretion. ** T valve tubing malfunction for 1 participant in the Bronchiectasis study and Sentec maintenance error for 1 participant in the COPD study. See Online Supplement for further detail. See legend of Table 3 and Online Supplement for further N value details

Participants were recruited between October 2015 and May 2017 and the CONSORT diagrams are shown in Fig. 1. The Neuromuscular/kyphoscoliosis study recruitment was stopped at 20 participants due to difficulty in recruitment. Participant characteristics are summarised in Table 2. The COPD group had higher smoking rates than the other two groups, and had similar severity of airflow obstruction as the Bronchiectasis group. One participant in the COPD study had a SpO_2_ of 87% at study baseline, all other participants had a SpO_2_ of ≥91%. All study baseline PtCO_2_ values were < 45 mmHg, with the exception of two participants in the Neuromuscular/kyphoscoliosis group.

**Table 2:** Participant characteristics and study baseline measurements

	Neuromuscular disease/ Kyphoscoliosis ***N*** = 20^**a**^	Bronchiectasis ***N*** = 24^**a**^	COPD ***N*** = 24^**a**^
**N (%)**			
Diagnosis	Neuromuscular disease: 18 Kyphoscoliosis: 2^**b**^	Bronchiectasis: 24 Cystic Fibrosis: 0	COPD: 24
Male	8 (40%)	7 (29%)	12 (50%)
Home NIV	4 (20%)	2 (8%)	1 (4%)
Home oxygen	1 (5%)	0 (0%)	0 (0%)
Smoking status	Total *N* = 18^**c**^	Total N = 23^**c**^	Total N = 18^**c**^
- Current	1 (6%)	0 (0%)	3 (17%)
- Ex	7 (39%)	3 (13%)	15 (83%)
- Never	10 (56%)	20 (87%)	0 (0%)
**Mean (SD)**			
Age (years)	52.2 (14.9)	63.0 (12.0)	69.4 (7.3)
BMI (kg/m^2^)	25.3 (7.2)	27.1 (4.7)	27.9 (4.8)
Smoking pack years	4.2 (9.1)	0.6 (2.2)	35.5 (22.9)
FEV_1_ percentage predicted (%)	57.6 (20.5)	69.9 (22.4)	65.7 (17.3)
FVC percentage predicted (%)	57.7 (20.5)	87.4 (21.5)	97.5 (17.9)
FEV_1_/FVC ratio (%)	83.0 (7.3)	64.6 (12.2)	53.5 (11.1)
PtCO_2_ (mmHg)	38.7 (4.8)	36.6 (3.4)	35.7 (3.5)^**d**^
- Min to max	27.8 to 48.6	29.3 to 42.8	29.8 to 42.1
Oxygen saturation (%)	95.7 (2.4)	96.3 (1.3)	95.4 (2.5)^**d**^
- Min to max	91 to 100	93 to 99	87 to 99
Respiratory rate (breaths/minute)	18.8 (4.5)	16.8 (3.2)	17.0 (3.9)
**Neuromuscular disease/kyphoscoliosis study entry criteria and diagnoses, N**			
Participants with SNIP < 95% range^**e**^	12	NA	NA
Participants with VC drop ≥10% sitting to lying^**f**^	13	NA	NA
Neuromuscular Diagnosis		NA	NA
- Charcot Marie Tooth	3		
- Facioscapulohumeral muscular dystrophy	2		
- Limb girdle muscular dystrophy	1		
- Motor neurone disease	5		
- Multiple sclerosis	1		
- Myotonic dystrophy	4		
- Phrenic nerve palsy	1		
- Tetraplegia	1		

*COPD* Chronic Obstructive Pulmonary Disease, *BMI* Body mass index, *FEV*_*1*_ Forced expiratory volume in 1 s, *FVC* Forced vital capacity, *NA* Not applicable, *NIV* Non-invasive ventilation, *PtCO*_*2*_ Transcutaneous partial pressure of carbon dioxide, *SNIP* Sniff nasal inspiratory pressure, *VC* Vital capacity (slow)

^**a**^ Unless otherwise stated

^**b**^ One kyphoscoliosis participant also had Ehlers-Danlos Syndrome, and meet both the spirometry and SNIP entry criteria for neuromuscular disease patients

^**c**^ Data unavailable from some participants. In the COPD study six participants did not report whether they were current or ex smokers, however all had pack year histories of at least 19 years

^**d**^*N* = 23, data unavailable due to SenTec failure, see Fig. 1 and Online Supplement for details

^**e**^Total N in which data was measured = 19

^**f**^Total N in which data was measured = 18

See Online Supplement for ethnicity and respiratory comorbidity data

### PtCO_2_

PtCO_2_ rose after the mask was applied in both interventions, returning to study baseline within 10 min of removal. At T = 0, (i.e. after placement and stabilisation of the mask, but prior to receiving the intervention) the average PtCO_2_ increase was at least 1.3 mmHg higher than the last PtCO_2_ measurement prior to mask placement (Table 3).

**Table 3:** Transcutaneous carbon dioxide outcomes

	Neuromuscular disease/ Kyphoscoliosis ***N*** = 20^**a**^	Bronchiectasis ***N*** = 24^**a**^	COPD ***N*** = 24^**a**^
**Change in PtCO**_**2**_**on placement of mask**^**b**^ **Mean (SD), mmHg**			
Intervention 1	1.8 (2.8)	1.3 (1.7)	1.3 (1.4)^**f**^
Intervention 2	1.3 (1.1)^**d**^	2.0 (1.4)^**f**^	1.8 (1.4)^**f**^
**PtCO**_**2**_**during air and oxygen interventions** **Mean (SD), mmHg**			
Oxygen T = 0	39.4 (4.2)^**d**^	38.5 (2.6)	37.0 (3.2)^**f**^
Oxygen T = 30	40.3 (4.1)^**d**^	39.6 (2.8)^**e**^	38.8 (3.5)^**f**^
Air T = 0	40.2 (5.6)	38.6 (2.7)^**f**^	37.3 (3.5)^**f**^
Air T = 30	39.7 (3.6)^**c**^	38.9 (2.9)^**f**^	37.7 (3.3)^**f**^
**Mixed linear model estimates**^**g**^**(95% CI), mmHg**			
Change at 30 min, oxygen minus air	0.2 (− 0.4 to 0.9) P = 0.40	0.5 (− 0.2 to 1.2) P = 0.18	1.3 (0.7 to 1.8) *P* < 0.001
Change over duration of intervention, oxygen minus air^**h**^	−0.07 (− 0.40 to 0.27) *P* = 0.70	0.4 (0.08 to 0.7) *P* = 0.012	1.3 (1.0 to 1.5) P < 0.001

*Air* Air intervention, *COPD* Chronic obstructive pulmonary disease, *Oxygen* Oxygen intervention, *PtCO*_*2*_ Transcutaneous partial pressure of carbon dioxide, *T = 0* Value taken at Time 0 min (i.e. following mask stabilisation and prior to start of intervention), *T = 30* Value taken at Time 30 min

^**a**^Unless otherwise stated

^**b**^Change is PtCO_2_ at T = 0 minus last recorded PtCO_2_ value prior to placement of mask

^**c**^*N* = 17

^**d**^*N* = 18

^**e**^*N* = 22

^**f**^*N* = 23

^**g**^ Mixed linear model values represent oxygen minus air change from T = 0

^**h**^Incorporated values are from T = 10, 20 and 30 min. The interaction between each time point (10, 20 and 30 min) was not significantly different, see Online Supplement for *P* values. N values for PtCO_2_ at each time point during the study were as follows:

Neuromuscular/Kyphoscoliosis study oxygen intervention/washout: *n* = 18 at T = 0 to T = 50, *n* = 17 at T = 60

Neuromuscular/Kyphoscoliosis study air intervention/washout: *n* = 20 at T = 0, *n* = 19 at T = 10, *n* = 18 at T = 20 and *n* = 17 at all other time points

Bronchiectasis study oxygen intervention/washout: *n* = 24 at T = 0 and T = 10, *n* = 23 at T = 20, *n* = 22 at all other time points

Bronchiectasis study air intervention/washout: n = 23 at T = 0 to T = 50 and *n* = 23 at T = 60

COPD oxygen and air interventions/washouts: *n* = 23 at all time points

See Online Supplement for further N value details

**Fig. 2:**
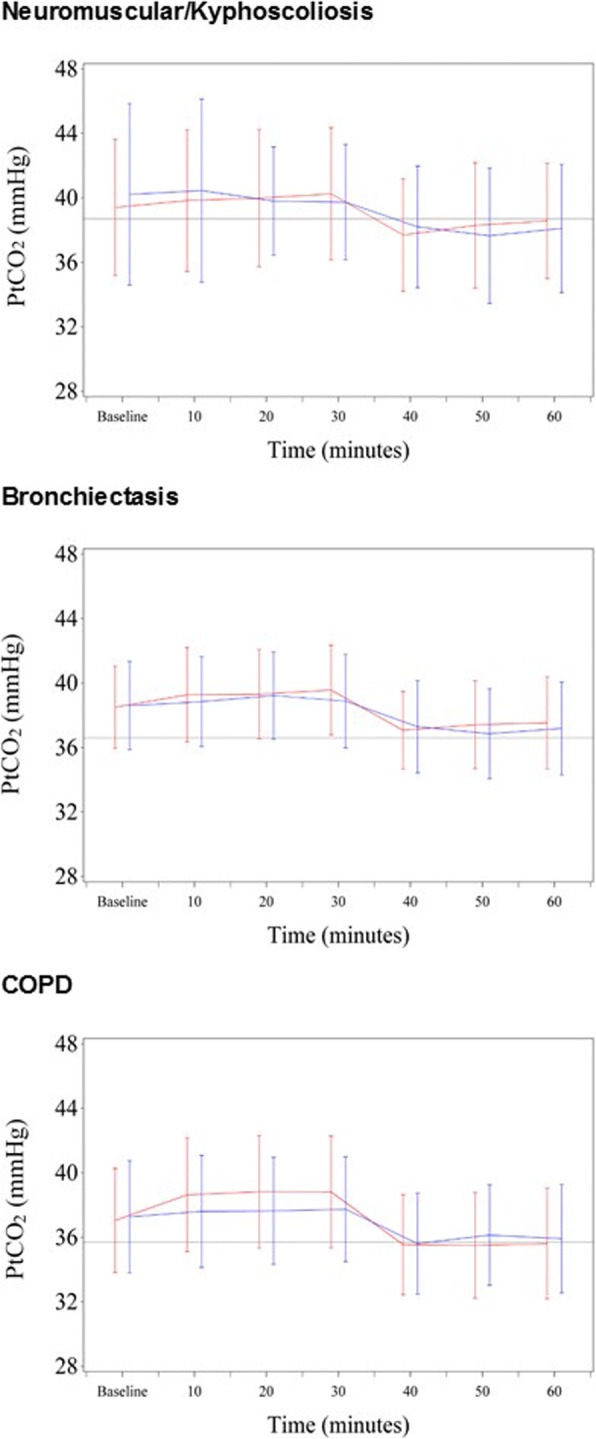
Transcutaneous partial pressure of carbon dioxide over time. COPD: Chronic obstructive pulmonary disease, PtCO_2_: Transcutaneous partial pressure of carbon dioxide. Blue lines represent oxygen intervention data, red represent air intervention data. Values are mean plus/minus 1SD. The solid grey line represents study baseline PtCO_2_ (prior to the start of the first intervention while breathing room air without a mask). T = 0 to T = 30 are measurements taken while wearing the study mask. T = 40 to T = 60 are washout measurements breathing room air, not wearing the study mask. See legend of Table 3 and Online Supplement for N value details

Figure 2 demonstrates PtCO_2_ over the course of the study (see Online Supplement for individual time point data). The difference (95% CI) in PtCO_2_ at 30 min between oxygen and room air, adjusted for T = 0 PtCO_2_, was 0.2 mmHg (− 0.4 to 0.9), *P* = 0.40; 0.5 mmHg (− 0.2 to 1.2), *P* = 0.18; and 1.3 mmHg (0.7 to 1.8), *P* < 0.001, in the Neuromuscular/kyphoscoliosis, Bronchiectasis and COPD participants respectively (Table 3). PtCO_2_ did not increase or decrease by ≥4 mmHg from T = 0 during the interventions, with the exception of one Bronchiectasis and one COPD participant, during the oxygen intervention only (increases of 4.8 mmHg and 4.7 mmHg respectively). The interaction terms between the time points (10, 20 and 30 min) were not significantly different. The mixed linear model estimates for the differences in PtCO_2_ across all time points and adjusted for T = 0 were slightly higher during the oxygen intervention compared to room air in the Bronchiectasis and COPD studies (Table 3). When compared to the Bronchiectasis participants, the COPD participants had a greater mean difference in PtCO_2_ adjusted for T = 0 between the oxygen and air interventions: 0.90 mmHg (95% CI 0.5 to 1.3), *P* < 0.001. There was no change to this estimate after incorporation of FEV_1_ percentage predicted as a potential confounder.

### Changes in other respiratory measures

Secondary outcomes are presented in Table 4. In the Bronchiectasis participants, the mean ETCO_2_ decreased by 1.0 mmHg during the oxygen intervention, compared with air. This was associated with a small increase in dead space (0.01 L) and VD/VT (0.03). In the COPD group the mean ETCO_2_ decreased by 1.1 mmHg, and this was associated with a small reduction in alveolar minute ventilation (0.21 L/min) and increase in VD/VT (0.023).

**Table 4:** Secondary outcomes

	Neuromuscular disease/ Kyphoscoliosis			Bronchiectasis			COPD		
	Oxy T = 0 Mean (SD) ***N*** = 18	Air T = 0 Mean (SD) ***N*** = 20	Estimate (95% CI) P value^**a**^	Oxy T = 0 Mean (SD) ***N*** = 24	Air T = 0 Mean (SD) ***N*** = 23	Estimate (95% CI) P value^**a**^	Oxy T = 0 Mean (SD) ***N*** = 24^**b**^	Air T = 0 Mean (SD) ***N*** = 24^**b**^	Estimate (95% CI) P value^**a**^
Minute ventilation (L/min)	6.8 (2.0)	6.3 (2.0)	0.17 (−0.26 to 0.59) *P* = 0.44	7.56 (2.49)	7.13 (1.88)	0.41 (−0.1 to 0.89) *P* = 0.09	8.08 (2.72)	8.03 (2.48)	−0.13 (− 0.55 to 0.29) *P* = 0.55
Respiratory rate (breaths/minute)	15.7 (5.1)	15.9 (5.6)	−0.01 (− 0.73 to 0.71) *P* = 0.98	15.5 (2.9)	14.7 (3.1)	0.4 (−0.4 to 1.2) *P* = 0.34	15.4 (5.0)	14.5 (4.4)	−0.3 (−1.0 to 0.3) *P* = 0.31
Tidal volume (L)	0.48 (0.21)	0.44 (0.2)	0.004 (−0.024 to 0.033) *P* = 0.77	0.50 (0.2)	0.50 (0.15)	0.01 (−0.03 to 0.05) *P* = 0.65	0.56 (0.18) ^**c**^	0.58 (0.17) ^**c**^	−0.003 (− 0.03 to 0.03) *P* = 0.82
Alveolar minute ventilation (L/min)	3.18 (1.28)	2.88 (1.33)	−0.03 (− 0.24 to 0.18) *P* = 0.78	3.26 (1.23)	3.10 (1.05)	−0.06 (− 0.26 to − 0.15) *P* = 0.58	3.16 (0.94) ^**c**^	3.15 (0.76) ^**c**^	−0.21 (− 0.38 to − 0.04) *P* = 0.014
Alveolar volume (L)	0.24 (0.15)	0.21 (0.13)	− 0.01 (− 0.023 to 0.01) P = 0.44	0.22 (0.10)	0.22 (0.09)	−0.02 (− 0.04 to 0.01) *P* = 0.14	0.23 (0.10) ^**c**^	0.24 (0.11) ^**c**^	− 0.01 (− 0.03 to 0.001) *P* = 0.065
ETCO_2_ (mmHg)	34.1 (2.9)	33.7 (3.4)	−0.20 (−1.0 to 0.60) *P* = 0.62	31.8 (3.6)	31.2 (4.4)	−1.0 (− 1.7 to − 0.3) *P* = 0.004	29.0 (3.9)	29.2 (4.0)	−1.1 (− 1.7 to − 0.5) *P* < 0.001
Volume of dead space (L)	0.24 (0.09)	0.23 (0.08)	0.012 (− 0.004 to 0.027) *P* = 0.13	0.28 (0.10)	0.28 (0.07)	0.01 (0.006 to 0.05) *P* = 0.011	0.33 (0.09) ^**c**^	0.33 (0.09) ^**c**^	0.009 (−0.007 to 0.03) *P* = 0.27
VD/VT	0.54 (0.11)	0.56 (0.09)	0.009 (−0.004 to 0.023) *P* = 0.17	0.57 (0.06)	0.57 (0.07)	0.03 (0.02 to 0.04) P < 0.001	0.60 (0.07) ^**c**^	0.59 (0.09) ^**c**^	0.023 (0.01 to 0.037) P < 0.001
Heart rate (beats per minute)	69.7 (13.8)	68.7 (13.3)	0.42 (−1.0 to 1.9) *P* = 0.56	73.7 (10.0)	74.9 (11.7)	−1.5 (−2.8 to −0.1) *P* = 0.036	71.9 (11.7) ^**c**^	69.9 (11.8)^**c**^	−3.3 (− 4.6 to − 2.1) P < 0.001

*Air* Air intervention, *COPD* Chronic obstructive pulmonary disease, *ETCO*_*2*_ End tidal carbon dioxide, *Oxy* Oxygen intervention, *VD/VT* Dead space to tidal volume ratio

^a^Mixed linear model results, oxygen minus air change from T = 0. All models are pooled estimates across all measurement times (10, 20 or 30 min) as the interaction between time point was not significantly different for any of the time points for any of the above outcomes, see Online Supplement for P values. See Online Supplement for data at individual time points and N value details

^b^ Unless otherwise stated

^c^*N* = 23

## Discussion

These randomised cross-over studies have shown that 50% oxygen for 30 min did not result in clinically significant increases in PtCO_2_ at 30 min in patients with stable COPD, Bronchiectasis or Neuromuscular disease/Kyphoscoliosis. In the patients with stable COPD the mean PtCO_2_ increase with high-concentration oxygen therapy was 1.3 mmHg; while this was statistically significant, the magnitude of the change is not of clinical significance. This is in contrast with the marked increases in PaCO_2_ seen in the setting of acute exacerbations of COPD [1, 5, 6]. This suggests that the results of all three studies in stable patients are unlikely to be generalisable to the use of oxygen therapy in the acute clinical setting.

To our knowledge there have been no RCTs investigating the effects of oxygen on PaCO_2_ in acutely unwell patients with neuromuscular disease, kyphoscoliosis or bronchiectasis. However the risk of oxygen-induced hypercapnia has been well established through RCTs comparing high-concentration and titrated oxygen regimens in patients with acute COPD exacerbations [6]. We undertook the current studies in patients with neuromuscular disease, kyphoscoliosis or bronchiectasis while stable, recognising that the results from stable patients in the laboratory setting may not translate to the clinical setting. We therefore conducted the study in COPD patients, as a comparator group in which clinically relevant oxygen-induced PaCO_2_ elevations have been demonstrated [1, 5, 6]. The study baseline SpO_2_ and PtCO_2_ values were comparable across all three studies, and FEV_1_ percentage predicted matching between the COPD and Bronchiectasis patients ensured recruitment of patients with similar physiological impairment in terms of airflow obstruction. Contrary to findings in the acute setting [1, 5, 6], oxygen administration did not result in a clinically significant change in PtCO_2_ in the stable COPD patients, indicating that the study model was not an appropriate method to detect the potential for oxygen-induced hypercapnia in patients with neuromuscular disease, kyphoscoliosis or bronchiectasis in clinical practice.

A number of factors may explain the minimal PtCO_2_ change in the COPD participants. Firstly, oxygen delivery was via a closed-circuit system, rather than standard masks used in clinical practice. This ensured precise fraction of inspired oxygen (FiO_2_) administration and allowed deadspace and ventilation measurement. However, this method of delivery may have affected participant’s responses to the interventions, particularly as breathing through the study mask consistently resulted in a small increase in PtCO_2_. Secondly, the studies were conducted in stable, rather than acutely unwell, patients. This allowed randomised cross-over trial design and meant that participants were more likely to tolerate study procedures. However, the physiological response to oxygen in stable patients may not translate to the acute setting. Previous studies investigating oxygen delivery to stable COPD patients have had variable results, ranging from no or small changes in mean PaCO_2_ or PtCO_2_ [24–31] to marked increases [23, 32–38]. Two studies have compared the effects of identical oxygen regimens in patients when having an acute exacerbation of their respiratory disease and when stable. Rudolf et al. found that an FiO_2_ of up to 0.28 for 1 h increased PaCO_2_ by 9, 15 and 31 mmHg compared with air in three patients with exacerbation of chronic respiratory failure [39]. However, the same oxygen regimen did not alter PaCO_2_ more than 3 mmHg when the same three patients were stable. Similarly, Aubier et al. found 30 min of oxygen via a mouthpiece increased average PaCO_2_ by 10.1 mmHg in 12 patients during a COPD exacerbation [25]. It increased by only 2.8 mmHg when the same patients were stable. The differences in response between acute and stable disease may relate to lower tidal volumes and/or a greater degree of hypoxic pulmonary vasoconstriction and ventilation/perfusion mismatch that occur in acute COPD exacerbation, which are further modified by oxygen therapy [40, 41]. Additionally, acutely unwell patients are more likely to have lower SpO_2_ levels and elevated PaCO_2_ levels. While hypercapnia and hypoxaemia are not necessarily prerequisites for oxygen-induced hypercapnia [38, 42], both have been associated with increased likelihood and magnitudes of oxygen-induced elevations in PaCO_2_ [10, 12, 23, 25, 33, 38]. In support of this, previous studies in stable COPD demonstrating significant oxygen-induced increases in PaCO_2_ have had participants with lower baseline blood oxygen levels [32, 34, 35, 38], and/or higher baseline PaCO_2_ values [23, 32–38] than the participants in the current three studies. Additionally, only one of the participants, from the COPD study, had a study baseline SpO_2_ of 87%. All other participants had saturations ≥91%, meaning they were well above the SpO_2_ level at which initiation of oxygen therapy is recommended in the acute clinical setting [2, 3].

Oxygen therapy has previously been demonstrated to increase VD/VT [10, 11, 23, 27, 35] and reduce ETCO_2_ [43]. However, caution is needed in interpreting the small increases in VD/VT and reductions in ETCO_2_ during the oxygen intervention in the Bronchiectasis and COPD studies. These values were recorded by CO_2_SMO and increased oxygen concentrations in the respiratory circuit could systematically decrease the displayed ETCO_2_ while still keeping it within the manufacturer’s error range of up to 5% (User manual Oct 10, 1997). This decrease could explain the small changes in VD/VT and ETCO_2_ observed.

There are a number of methodological issues relevant to interpretation of the study findings. Transcutaneous monitoring was used as a surrogate for PaCO_2_ by arterial blood gas (ABG). ABGs and capillary blood gas sampling were not used to measure PaCO_2_ during the interventions as they do not provide continuous measurement and cause discomfort. Additionally, ABG sampling carries risk of ischaemia. Our study outcome measures were change in PtCO_2_ over time, which the SenTec has been demonstrated to accurately determine [44, 45], with an estimate of bias for change in PtCO_2_ of − 0.03 mmHg (95% CI − 0.44 to 0.38) *p* = 0.89 when compared to arterialised blood gas values in COPD patients [45]. Only 20 participants were recruited to the Neuromuscular/kyphoscoliosis study, however this did not affect the power to detect a difference in PtCO_2_ between the interventions, given the SD was lower than that used for sample size calculation.

## Conclusion

Delivery of 50% oxygen for 30 min did not result in a clinically significant increase in PtCO_2_ in stable outpatients with neuromuscular disease, kyphoscoliosis, bronchiectasis or COPD. This indicates the model used is an inappropriate method for evaluating the risks of oxygen-induced hypercapnia in the acute clinical setting and highlights the limitations of interpreting results from studies in stable patients in the laboratory setting. It is recommended that future studies into the risks of oxygen-induced hypercapnia are undertaken through comparison of high-concentration oxygen to titrated oxygen in the acute respiratory illnesses that complicate neuromuscular disease, kyphoscoliosis and bronchiectasis. In the interim, current evidence of the potential for oxygen-induced hypercapnia to occur across a range of respiratory conditions [6, 8, 9, 12] supports guideline recommendations to titrate oxygen therapy in all patients to avoid the risks of hyperoxaemia as well as hypoxaemia.

## Supplementary information

**Additional file 1.**

## Data Availability

The datasets used and/or analysed during the current study are available from the corresponding author on reasonable request.

## Abbreviations

BMI: Body Mass Index
COPD: Chronic Obstructive Pulmonary Disease
CT: Computerised Tomography
ETCO_2_: End Tidal Carbon Dioxide
FEV_1_: Forced Expiratory Volume in 1 s
FiO_2_: Fraction of Inspired Oxygen
FVC: Forced Vital Capacity
MRINZ: Medical Research Institute of New Zealand
PaCO_2_: Arterial Partial Pressure of Carbon Dioxide
PtCO_2_: Transcutaneous Partial Pressure of Carbon Dioxide
RCT: Randomised Controlled Trial
SNIP: Sniff Nasal Inspiratory Pressure
SpO_2_: Oxygen Saturation
VC: Vital Capacity (slow)
VD/VT: Ratio of Dead Space to Tidal Volume
