# Supplementary material for: The effect of 50% oxygen on PtCO2 in patients with stable COPD, bronchiectasis, and neuromuscular disease or kyphoscoliosis: randomised cross-over trials
Source: BMC Pulm Med. 2020 May 7;20:125. doi: 10.1186/s12890-020-1132-z (PMC7203892; doi:10.1186/s12890-020-1132-z)
Supplement: Supplementary file 1 — Additional file 1. [file 12890_2020_1132_MOESM1_ESM.docx]

**SUPPLEMENTARY INFORMATION**

**The effect of 50% oxygen on PtCO_2_ in patients with stable COPD, bronchiectasis, and neuromuscular disease or kyphoscoliosis: randomised cross-over trials**

Janine Pilcher,^1,2,3^ Darmiga Thayabaran,^1,2^ Stefan Ebmeier,^1,2^ Mathew Williams,^1^

Geraldine Back,^2^ Hamish Collie,^2^ Michael Richards,^1,2^ Sue Bibby,^1,2^ Ruth Semprini,^1,2^

Mark Weatherall^4^ and Richard Beasley^1,2^

^1^Medical Research Institute of New Zealand, Wellington, New Zealand

^2^Capital & Coast District Health Board, Wellington, New Zealand

^3^Victoria University of Wellington, Wellington, New Zealand

^4^University of Otago, Wellington, New Zealand

**Contact:**

Professor Richard Beasley

Medical Research Institute of New Zealand

Private Bag 7902, Wellington 6242, New Zealand

Telephone: +64-4-805 0230

Facsimile: +64-4-389 5707

Email: Richard.beasley@mrinz.ac.nz

**Appendix S1. ADDITIONAL METHODOLOGY DETAILS**

**A. REGISTRATION AND APPROVAL**

Each trial was prospectively registered on the Australian New Zealand Clinical Trials Registry (ANZCTR) website and had prospective Health and Disability Ethics Committee approval, see Table S1).

**B. ADDITIONAL STUDY PROCEDURE DETAIL**

**Participant position**

The study was conducted while the participants were at rest and sitting at approximately 90 degrees.

**Spirometry and Sniff Nasal Inspiratory Pressure (SNIP)**

Spirometry was performed to ATS/ERS criteria^1^ using a handheld spirometer (Masterscreen non-heated pneumotach, CareFusion Germany 234 GmbH, Leibnizstrasse 7, 97204 Hoechberg, Germany).

SNIP testing (Neuromuscular disease participants only) was performed on a handheld device (MicroRPM CareFusion Germany 234 GmbH, Leibnizstrasse 7, 97204 Hoechberg, Germany).

**Mask stabilisation**

The mask was fitted while the participant was seated comfortably. Small, medium and large masks were available and fitted as appropriate. Once the mask was fitted with no evidence of leaks around the seal the participant breathed room air from the mask. Transcutaneous partial pressure of carbon dioxide (PtCO_2_) was recorded at 4 minutes and 5 minutes after fitting. If the PtCO_2_ values were within 1mmHg of each other then the T=0 measures were taken and the Intervention commenced. If values were not within 1mmHg of each other the stabilisation period was continued until two measures taken 1 minute apart were within 1mmHg.

**Washout period length**

The washout period of at least 30 minutes is based on work by Rudolf et al which found arterial partial pressure of carbon dioxide (PaCO_2_) approximately returned to baseline at 30 minutes following 60 minutes of oxygen administration.^2^ However should patients not return to within 4mmHg of their Intervention 1 baseline measurement after mask stabilisation for Intervention 2, the investigator extended the washout period until they did, for up to 20 minutes.

**Data calculations**

Tidal volume = [minute ventilation] / [respiratory rate]

Dead space volume = [tidal volume] X [VD/VT*]

Alveolar volume= [tidal volume] – [dead space volume]

Alveolar minute ventilation= [alveolar volume] X [respiratory rate]

Values in red recorded from CO_2_SMO.

*VD/VT: dead space to tidal volume ratio, obtained via CO_2_SMO calculation after input into the CO_2_SMO of simultaneously measured PtCO_2_ value from the SenTec.

**C. SENTEC USE**

**Equipment details and settings**

The SenTec was used with V-Sign Sensors, attached at the earlobe with single use clips. The arteriolisation temperature was set at 42 degrees.

**Maintenance and calibration**

Prior to each use the SenTec probe was checked for damage and underwent system calibration. Maintenance was as per SenTec manufacturer’s instructions (General (HB-005771-h) and Technical (HB-005752-g) manuals). Probe membranes were replaced every 28 days. An exception to this was a period where the SenTec had both quarterly maintenance and membrane replacement overdue due to Investigator error. Three participants were recruited during this time. It resulted in withdrawal of one Chronic Obstructive Pulmonary Disease (COPD) study participant during their study visit when it was realised data values were inaccurate, and removal of SenTec data from the dataset (see Section 2). Prior to this one participant in the COPD study and one in the Neuromuscular disease/Kyphoscoliosis study had data recorded when the procedures were overdue. Data were retained for analysis based on minor probe drift on review of subsequent drift corrected data, which suggested ongoing of probe accuracy.

**Stabilisation**

To ensure that the probe had stabilised prior to any data collection there was a stabilisation period following placement of the probe on the participant’s ear. At 28 minutes and 30 minutes the PtCO_2_ was recorded. If the values were within 1 mmHg of each other the study commenced. If not, stabilisation was continued until values were within 1mmHg of each other over a 2-minute period. The time period for stabilisation was based on previous work assessing time to probe stabilisation.^3,4^

**D. MAUFACTURER DETAILS**

Further detail regarding SAS 9.4 can be found at:

https://www.sas.com/en_us/software/sas9.html

Further detail regarding Hans Rudolf (supplier of Douglas bags) can be found at: http://www.rudolphkc.com

Further detail regarding Respironics (supplier of Full-face positive pressure masks) can be found at: https://www.philips.co.nz/healthcare/solutions/sleep-and-respiratory-care

**Appendix S2. N values**

**Neuromuscular disease/Kyphoscoliosis:**

The following participants withdrew due to inability to tolerate the mask:

- 1 Participant : Withdrew from study after T=0 Intervention 1, all subsequent data points not collected.
- 1 Participant: Withdrew from study after T=10 Intervention 1, all subsequent data points not collected.
- 1 Participant: Withdrew from Intervention 1 after T=20 due to cough, resulting in Intervention 1 T=30 and washout data not being collected.

Individual data unavailable:

- 1 Participant: at T=60 during Intervention 1 data not recorded as required restroom.

**Bronchiectasis:**

The following participant withdrew due to inability to tolerate the mask:

- 1 Participant: Withdrew from study after T=20 Intervention 1, all subsequent data points not collected.

Individual data unavailable:

- 1 Participant: at T=10 during Intervention 1 data not recorded as tubing failed, all subsequent data points for Intervention 1 not collected.
- 1 Participant: At T=60 during Intervention 1 earclip for SenTec disconnected with no SenTec data.*

**COPD:**

Participant data withdrawal:

- 1 Participant: all SenTec data had to be discarded due to SenTec maintenance error. No SenTec data was used.* CO_2_SMO data** was recorded until T=10 min for Intervention 2 only, when participant withdrawn based on identification of inability to rely on SenTec data.

Individual data unavailable:

- 1 Participant: at T=10 during Intervention 1 there was a mask leak and resulting CO_2_SMO inaccuracy. No CO_2_SMO data** was used for this time point.
- 1 Participant: at T=20 during Intervention 2 the CO_2_SMO did not report VD/VT. This meant that volume of dead space, alveolar minute ventilation and alveolar volume also could not be calculated.
- 1 Participant: at T=30 during Intervention 2 heart rate was not recorded due to Investigator error.

*SenTec data: PtCO_2_ and heart rate. Note VD/VT measurement requires PtCO_2_ to be available from SenTec. Note calculation of volume of dead space, alveolar minute ventilation and alveolar volume requires PtCO_2_ to be available from SenTec.

** CO_2_SMO data: End tidal carbon dioxide (ETCO_2_), minute ventilation, VD/VT and respiratory rate. Note calculation of tidal volume, volume of dead space, alveolar minute ventilation and alveolar volume require data from CO_2_SMO.

**Appendix S3. ADDITIONAL RESULTS**

**A. ADDITIONAL BASELINE VALUES**

Tables S2a and S2b contain additional baseline data.

**B. OXYGEN SATURATION VALUES**

On visual inspection of the uploaded SenTec oxygen saturation data there was no evidence of rebound hypoxia following the oxygen intervention.

**C. INDIVIDUAL TIME POINT DATA**

Individual time point data are presented in Tables S3a to S3l.

**D. TIME-TREATMENT INTERACTION TERMS**

Time-treatment interaction term P values are presented in Table S4.

**Appendix S4. TABLES**

**Table S1: Ethical approval and trial registration details**

| **Study** | **Registration Number** | **Ethical Approval Details** |
| --- | --- | --- |
| Neuromuscular disease/ Kyphoscoliosis | ACTRN12615000970549 | Submission number: CEN/11/11/065  Committee: Central Health and Disability Ethics Committee New Zealand |
| Bronchiectasis | ACTRN12615000971538 | Submission number: CEN/11/12/075  Committee: Central Health and Disability Ethics Committee New Zealand |
| COPD | ACTRN12615001056583 | Submission number: 13/STH/200  Committee: Southern Health and Disability Ethics Committee New Zealand |

**Table S2: Participant ethnicity and respiratory comorbidities**

|  | **Neuromuscular disease/ Kyphoscoliosis**  **N=20** | **Bronchiectasis**  **N=24** | **COPD**  **N=24** |
| --- | --- | --- | --- |
| Other respiratory diagnoses, N |  |  |  |
| - Asthma | 2 | 15 | 8 |
| - Sleep apnoea | 3 | 2 | 1 |
| Ethnicity, N   - NZ European - Māori - Chinese - Samoan - Indian - Other | 16  0  0  1  0  3 | 18  1  2  1  0  2 | 19  3  0  0  1  1 |

**Table S3a: Neuromuscular disease/Kyphoscoliosis Study: PtCO_2_**

|  | | **Time point**  **(min)** | **N** | **PtCO_2_**  **Mean (SD)** |
| --- | --- | --- | --- | --- |
| **Oxygen Intervention** | Immediately prior to intervention | 0 | 18 | 39.4 (4.2) |
|  | Intervention | 10 | 18 | 39.8 (4.4) |
|  |  | 20 | 18 | 40 (4.3) |
|  |  | 30 | 18 | 40.3 (4.1) |
|  | Washout | 40 | 18 | 37.7 (3.5) |
|  |  | 50 | 18 | 38.3 (3.9) |
|  |  | 60 | 17 | 38.6 (3.6) |
| **Air Intervention** | Immediately prior to intervention | 0 | 20 | 40.2 (5.6) |
|  | Intervention | 10 | 19 | 40.5 (5.7) |
|  |  | 20 | 18 | 39.8 (3.4) |
|  |  | 30 | 17 | 39.7 (3.6) |
|  | Washout | 40 | 17 | 38.2 (3.8) |
|  |  | 50 | 17 | 37.7 (4.2) |
|  |  | 60 | 17 | 38.1 (4.0) |
| **Difference from T=0, oxygen minus air** | Intervention | 10 | 18 | -0.1 (1.2) |
|  |  | 20 | 18 | -0.3 (1.4) |
|  |  | 30 | 17 | 0.2 (1.3) |
|  | Washout | 40 | 17 | -0.9 (2.0) |
|  |  | 50 | 17 | 0.3 (2.2) |
|  |  | 60 | 16 | -0.6 (2.6) |

PtCO_2_: Transcutaneous partial pressure of carbon dioxide. Grey cells are washout periods during which the participant breathed room air without a mask.

**Table S3b: Neuromuscular disease/Kyphoscoliosis Study: Minute ventilation, respiratory rate and tidal volume**

|  | **Time point**  **(min)** | **N** | **Mean (SD)** | | |
| --- | --- | --- | --- | --- | --- |
|  |  |  | **Minute ventilation**  **(L/min)** | **Respiratory rate**  **(breaths per minute)** | **Tidal Volume**  **(L)** |
| **Oxygen intervention** | 0 | 18 | 6.8 (2.0) | 15.7 (5.1) | 0.48 (0.21) |
|  | 10 | 18 | 6.6 (2.1) | 15.9 (4.3) | 0.45 (0.20) |
|  | 20 | 18 | 6.8 (1.7) | 15.3 (5.9) | 0.48 (0.14) |
|  | 30 | 18 | 6.9 (2.1) | 15.2 (4.6) | 0.48 (0.17) |
| **Air intervention** | 0 | 20 | 6.3 (2.0) | 15.9 (5.6) | 0.44 (0.20) |
|  | 10 | 19 | 6.3 (1.8) | 16.4 (6.2) | 0.43 (0.16) |
|  | 20 | 18 | 6.6 (1.7) | 15.4 (4.5) | 0.45 (0.15) |
|  | 30 | 17 | 6.3 (1.7) | 15.4 (4.8) | 0.46 (0.20) |
| **Difference from T=0, oxygen minus air interventions** | 10 | 18 | -0.2 (1.3) | 0.4 (2.4) | -0.02 (0.11) |
|  | 20 | 18 | 0.0 (2.1) | -0.1 (3.3) | 0.0 (0.10) |
|  | 30 | 17 | 0.3 (2.1) | -0.3 (3.5) | 0.0 (0.13) |

**Table S3c: Neuromuscular disease/Kyphoscoliosis Study: Alveolar minute ventilation and alveolar volume**

|  | **Time point**  **(min)** | **N** | **Mean (SD)** | |
| --- | --- | --- | --- | --- |
|  |  |  | **Alveolar minute ventilation**  **(L/min)** | **Alveolar volume**  **(L)** |
| **Oxygen intervention** | 0 | 18 | 3.2 (1.3) | 0.24 (0.15) |
|  | 10 | 18 | 2.9 (1.2) | 0.20 (0.12) |
|  | 20 | 18 | 3.0 (1.0) | 0.21 (0.10) |
|  | 30 | 18 | 2.9 (1.1) | 0.21 (0.10) |
| **Air intervention** | 0 | 20 | 2.9 (1.3) | 0.21 (0.13) |
|  | 10 | 19 | 2.8 (1.0) | 0.19 (0.09) |
|  | 20 | 18 | 3.0 (1.1) | 0.21 (0.10) |
|  | 30 | 17 | 2.8 (1.1) | 0.21 (0.12) |
| **Difference from T=0, oxygen minus air interventions** | 10 | 18 | -0.1 (0.7) | -0.02 (0.05) |
|  | 20 | 18 | -0.2 (1.1) | -0.01 (0.07) |
|  | 30 | 17 | 0.0 (1.1) | -0.01 (0.09) |

**Table S3d: Neuromuscular disease/Kyphoscoliosis Study: ETCO_2_, volume of dead space, VD/VT and heart rate**

|  | **Time point**  **(Min)** | **N** | **Mean (SD)** | | | |
| --- | --- | --- | --- | --- | --- | --- |
|  |  |  | **ETCO_2_**  **(mmHg)** | **Volume of dead space**  **(L)** | **VD/VT** | **Heart rate**  **(Beats/minute)** |
| **Oxygen Intervention** | 0 | 18 | 34.1 (2.9) | 0.24 (0.09) | 0.54 (0.11) | 69.7 (13.8) |
|  | 10 | 18 | 33.1 (3.2) | 0.25 (0.10) | 0.57 (0.09) | 69.6 (13.6) |
|  | 20 | 18 | 33.6 (3.7) | 0.26 (0.07) | 0.56 (0.10) | 69.4 (14.5) |
|  | 30 | 18 | 32.6 (2.8) | 0.27 (0.09) | 0.57 (0.09) | 67.9 (13.9) |
| **Air Intervention** | 0 | 20 | 33.7 (3.4) | 0.23 (0.08) | 0.56 (0.09) | 68.7 (13.3) |
|  | 10 | 19 | 32.4 (4.6) | 0.24 (0.07) | 0.57 (0.08) | 66.2 (10.1) |
|  | 20 | 18 | 33.4 (2.4) | 0.24 (0.06) | 0.55 (0.08) | 67.1 (10.4) |
|  | 30 | 17 | 33.4 (3.7) | 0.24 (0.08) | 0.56 (0.07) | 69.1 (11.2) |
| **Difference from T=0, oxygen minus air interventions** | 10 | 18 | -0.4 (3.5) | 0.00 (0.07) | 0.02 (0.04) | 1.2 (6.0) |
|  | 20 | 18 | -0.2 (3.8) | 0.01 (0.05) | 0.01 (0.07) | 1.1 (6.1) |
|  | 30 | 17 | -0.9 (3.7) | 0.02 (0.06) | 0.01 (0.07) | -1.8 (6.0) |

ETCO_2_: End tidal carbon dioxide, VD/VT: Dead space to tidal volume ratio

**Table S3e: Bronchiectasis Study: PtCO_2_**

|  | | **Time point**  **(min)** | **N** | **PtCO_2_**  **Mean (SD)** |
| --- | --- | --- | --- | --- |
| **Oxygen Intervention** | Immediately prior to intervention | 0 | 24 | 38.5 (2.6) |
|  | Intervention | 10 | 24 | 39.3 (2.9) |
|  |  | 20 | 23 | 39.3 (2.8) |
|  |  | 30 | 22 | 39.6 (2.8) |
|  | Washout | 40 | 22 | 37.1 (2.4) |
|  |  | 50 | 22 | 37.4 (2.7) |
|  |  | 60 | 22 | 37.5 (2.8) |
| **Air Intervention** | Immediately prior to intervention | 0 | 23 | 38.6 (2.7) |
|  | Intervention | 10 | 23 | 38.8 (2.8) |
|  |  | 20 | 23 | 39.2 (2.7) |
|  |  | 30 | 23 | 38.9 (2.9) |
|  | Washout | 40 | 23 | 37.3 (2.9) |
|  |  | 50 | 23 | 36.9 (2.8) |
|  |  | 60 | 22 | 37.2 (2.9) |
| **Difference from T=0, oxygen minus air** | Intervention | 10 | 23 | 0.6 (1.2) |
|  |  | 20 | 22 | 0.0 (1.6) |
|  |  | 30 | 22 | 0.5 (1.7) |
|  | Washout | 40 | 22 | -0.5 (1.8) |
|  |  | 50 | 22 | 0.3 (2.3) |
|  |  | 60 | 21 | 0.1 (2.4) |

PtCO_2_: Transcutaneous partial pressure of carbon dioxide. Grey cells are washout periods during which the participant breathed room air without a mask.

**Table S3f: Bronchiectasis Study: Minute ventilation, respiratory rate and tidal volume**

|  | **Time point**  **(min)** | **N** | **Mean (SD)** | | |
| --- | --- | --- | --- | --- | --- |
|  |  |  | **Minute ventilation**  **(L/min)** | **Respiratory rate**  **(breaths per minute)** | **Tidal Volume**  **(L)** |
| **Oxygen intervention** | 0 | 24 | 7.56 (2.49) | 15.5 (2.9) | 0.50 (0.20) |
|  | 10 | 24 | 7.70 (2.09) | 15.5 (2.9) | 0.52 (0.19) |
|  | 20 | 23 | 7.73 (2.18) | 15.3 (2.7) | 0.52 (0.16) |
|  | 30 | 22 | 8.01 (2.60) | 16.4 (3.4) | 0.50 (0.17) |
| **Air intervention** | 0 | 23 | 7.13 (1.88) | 14.7 (3.1) | 0.50 (0.15) |
|  | 10 | 23 | 7.07 (1.55) | 14.4 (3.4) | 0.54 (0.25) |
|  | 20 | 23 | 7.60 (2.11) | 16.0 (3.1) | 0.49 (0.15) |
|  | 30 | 23 | 6.74 (2.16) | 15.0 (3.9) | 0.47 (0.18) |
| **Difference from T=0, oxygen minus air interventions** | 10 | 23 | 0.04 (1.83) | 0.1 (3.5) | -0.02 (0.26) |
|  | 20 | 22 | -0.55 (2.83) | -1.3 (5.3) | 0.00 (0.21) |
|  | 30 | 22 | 0.66 (2.31) | 0.7 (4.3) | 0.01 (0.21) |

**Table S3g: Bronchiectasis Study: Alveolar minute ventilation and alveolar volume**

|  | **Time point**  **(min)** | **N** | **Mean (SD)** | |
| --- | --- | --- | --- | --- |
|  |  |  | **Alveolar minute ventilation**  **(L/min)** | **Alveolar volume**  **(L)** |
| **Oxygen intervention** | 0 | 24 | 3.26 (1.23) | 0.22 (0.10) |
|  | 10 | 24 | 3.07 (0.97) | 0.21 (0.09) |
|  | 20 | 23 | 3.07 (0.95) | 0.21 (0.07) |
|  | 30 | 22 | 3.11 (1.06) | 0.20 (0.08) |
| **Air intervention** | 0 | 23 | 3.10 (1.05) | 0.22 (0.09) |
|  | 10 | 23 | 3.11 (0.90) | 0.25 (0.16) |
|  | 20 | 23 | 3.15 (1.04) | 0.21 (0.09) |
|  | 30 | 23 | 2.87 (1.1) | 0.21 (0.11) |
| **Difference from T=0, oxygen minus air interventions** | 10 | 23 | -0.28 (0.98) | -0.04 (0.15) |
|  | 20 | 22 | -0.36 (1.18) | -0.01 (0.10) |
|  | 30 | 22 | 0.02 (1.21) | -0.01 (0.11) |

**Table S3h: Bronchiectasis Study: ETCO_2_, volume of dead space, VD/VT and heart rate**

|  | **Time point**  **(Min)** | **N** | **Mean (SD)** | | | |
| --- | --- | --- | --- | --- | --- | --- |
|  |  |  | **ETCO_2_**  **(mmHg)** | **Volume of dead space**  **(L)** | **VD/VT** | **Heart rate**  **(Beats/minute)** |
| **Oxygen Intervention** | 0 | 24 | 31.8 (3.6) | 0.28 (0.10) | 0.57 (0.06) | 73.7 (10.0) |
|  | 10 | 24 | 30.1 (3.0) | 0.31 (0.11) | 0.61 (0.05) | 68.8 (9.8) |
|  | 20 | 23 | 29.8 (2.9) | 0.31 (0.09) | 0.61 (0.05) | 69.6 (9.5) |
|  | 30 | 22 | 29.8 (2.7) | 0.30 (0.10) | 0.61 (0.05) | 70.2 (10.1) |
| **Air Intervention** | 0 | 23 | 31.2 (4.4) | 0.28 (0.07) | 0.57 (0.06) | 74.9 (11.7) |
|  | 10 | 23 | 31.0 (3.9) | 0.29 (0.10) | 0.56 (0.08) | 72.5 (9.3) |
|  | 20 | 23 | 31.0 (3.6) | 0.28 (0.07) | 0.59 (0.06) | 72.2 (11.1) |
|  | 30 | 23 | 30.0 (3.9) | 0.26 (0.08) | 0.58 (0.08) | 72.2 (10.7) |
| **Difference from T=0, oxygen minus air interventions** | 10 | 23 | -1.3 (3.4) | 0.01 (0.12) | 0.04 (0.05) | -2.3 (6.0) |
|  | 20 | 22 | -1.6 (4.2) | 0.01 (0.12) | 0.02 (0.04) | -0.6 (8.5) |
|  | 30 | 22 | -0.9 (3.5) | 0.03 (0.11) | 0.03 (0.05) | 0.0 (6.7) |

ETCO_2_: End tidal carbon dioxide, VD/VT: Dead space to tidal volume ratio

**Table S3i: COPD Study: PtCO_2_**

|  | | **Time point**  **(min)** | **N** | **PtCO_2_**  **Mean (SD)** |
| --- | --- | --- | --- | --- |
| **Oxygen Intervention** | Immediately prior to intervention | 0 | 23 | 37.0 (3.2) |
|  | Intervention | 10 | 23 | 38.6 (3.5) |
|  |  | 20 | 23 | 38.8 (3.5) |
|  |  | 30 | 23 | 38.8 (3.5) |
|  | Washout | 40 | 23 | 35.5 (3.1) |
|  |  | 50 | 23 | 35.5 (3.3) |
|  |  | 60 | 23 | 35.6 (3.4) |
| **Air Intervention** | Immediately prior to intervention | 0 | 23 | 37.3 (3.5) |
|  | Intervention | 10 | 23 | 37.6 (3.5) |
|  |  | 20 | 23 | 37.6 (3.3) |
|  |  | 30 | 23 | 37.7 (3.3) |
|  | Washout | 40 | 23 | 35.6 (3.1) |
|  |  | 50 | 23 | 36.1 (3.1) |
|  |  | 60 | 23 | 35.9 (3.4) |
| **Difference from T=0, oxygen minus air** | Intervention | 10 | 23 | 1.2 (1.4) |
|  |  | 20 | 23 | 1.4 (1.5) |
|  |  | 30 | 23 | 1.3 (1.3) |
|  | Washout | 40 | 23 | 0.2 (1.8) |
|  |  | 50 | 23 | -0.4 (2.0) |
|  |  | 60 | 23 | -0.1 (2.4) |

COPD: Chronic obstructive pulmonary disease, PtCO_2_: Transcutaneous partial pressure of carbon dioxide.

Grey cells are washout periods during which the participant breathed room air without a mask.

**Table S3j: COPD Study: Minute ventilation, respiratory rate and tidal volume**

|  | **Time point**  **(min)** | **N*** | **Mean (SD)** | | |
| --- | --- | --- | --- | --- | --- |
|  |  |  | **Minute ventilation**  **(L/min)** | **Respiratory rate**  **(breaths per minute)** | **Tidal Volume**  **(L)** |
| **Oxygen intervention** | 0 | 24 | 8.08 (2.72) | 15.4 (5.0) | 0.56 (0.18)** |
|  | 10 | 24 | 7.80 (2.86) | 16.2 (5.2) | 0.51 (0.20)** |
|  | 20 | 24 | 8.26 (3.15) | 16.7 (4.6) | 0.53 (0.17)*** |
|  | 30 | 24 | 7.72 (2.98) | 15.2 (4.2) | 0.52 (0.19)** |
| **Air intervention** | 0 | 24 | 8.03 (2.48) | 14.5 (4.4) | 0.58 (0.17)** |
|  | 10 | 23 | 7.84 (2.77) | 15.7 (4.7) | 0.53 (0.19)*** |
|  | 20 | 23 | 7.90 (2.49) | 15.8 (3.9) | 0.52 (0.15) |
|  | 30 | 23 | 8.36 (3.14) | 15.6 (4.4) | 0.54 (0.16) |
| **Difference from T=0, oxygen minus air interventions** | 10 | 23 | 0.03 (1.62) | -0.3 (2.5) | 0.02 (0.16)*** |
|  | 20 | 23 | 0.26 (2.47) | 0.0 (2.8) | 0.01 (0.19)*** |
|  | 30 | 23 | -0.70 (2.19) | -1.2 (3.2) | 0.00 (0.18) |

COPD: Chronic obstructive pulmonary disease, * Unless otherwise indicated, ** N=23,

*** N=22.

**Table S3k: COPD Study: Alveolar minute ventilation and alveolar volume**

|  | **Time point**  **(min)** | **N** | **Mean (SD)** | |
| --- | --- | --- | --- | --- |
|  |  |  | **Alveolar minute ventilation**  **(L/min)** | **Alveolar volume**  **(L)** |
| **Oxygen intervention** | 0 | 23 | 3.16 (0.94) | 0.23 (0.10) |
|  | 10 | 23 | 2.80 (0.92) | 0.19 (0.10) |
|  | 20 | 22 | 3.04 (0.87) | 0.20 (0.09) |
|  | 30 | 23 | 2.81 (0.96) | 0.20 (0.10) |
| **Air intervention** | 0 | 23 | 3.15 (0.76) | 0.24 (0.11) |
|  | 10 | 22 | 2.98 (0.94) | 0.21 (0.12) |
|  | 20 | 23 | 3.09 (0.94) | 0.21 (0.09) |
|  | 30 | 23 | 3.13 (0.89) | 0.21 (0.08) |
| **Difference from T=0, oxygen minus air interventions** | 10 | 22 | -0.12 (0.96) | 0.00 (0.09) |
|  | 20 | 22 | -0.17 (1.38) | -0.01 (0.11) |
|  | 30 | 23 | -0.32 (1.09) | 0.00 (0.10) |

COPD: Chronic obstructive pulmonary disease

**Table S3l: COPD Study: ETCO_2_, volume of dead space, VD/VT and heart rate**

|  | **Time point**  **(Min)** | **N*** | **Mean (SD)** | | | |
| --- | --- | --- | --- | --- | --- | --- |
|  |  |  | **ETCO_2_**  **(mmHg)** | **Volume of dead space**  **(L)** | **VD/VT** | **Heart rate**  **(Beats/minute)** |
| **Oxygen Intervention** | 0 | 23 | 29.0 (3.9)** | 0.33 (0.09) | 0.60 (0.07) | 71.9 (11.7) |
|  | 10 | 23 | 28.1 (3.7)** | 0.32 (0.11) | 0.63 (0.09) | 69.8 (12.1) |
|  | 20 | 23 | 28.6 (3.8)** | 0.33 (0.10)  *** | 0.63 (0.07)  *** | 68.0 (12.2) |
|  | 30 | 23 | 27.9 (3.7)** | 0.33 (0.11) | 0.63 (0.07) | 68.9 (11.7)*** |
| **Air Intervention** | 0 | 23 | 29.2 (4.0)** | 0.33 (0.09) | 0.59 (0.09) | 69.9 (11.8) |
|  | 10 | 23 | 29.3 (4.0) | 0.31 (0.10)  *** | 0.61 (0.08)  *** | 71.2 (10.5) |
|  | 20 | 23 | 29.6 (3.7) | 0.31 (0.08) | 0.60 (0.07) | 71.0 (11.2) |
|  | 30 | 23 | 29.3 (4.1) | 0.33 (0.10) | 0.61 (0.07) | 69.8 (11.5) |
| **Difference from T=0, oxygen minus air interventions** | 10 | 23 | -0.9 (3.7) | - 1. (0.08)   *** | 0.01 (0.09)  *** | -3.4 (6.5) |
|  | 20 | 23 | -0.7 (4.4) | 0.02 (0.10)  *** | 0.02 (0.09)  *** | -5.0 (6.9) |
|  | 30 | 23 | -1.2 (4.5) | 0.00 (0.09) | 0.01 (0.08) | -3.9 (6.3)*** |

COPD: Chronic obstructive pulmonary disease, ETCO_2_: End tidal carbon dioxide, VD/VT: Dead space to tidal volume ratio, * Unless otherwise indicated, ** N=24, ***N=22.

**Table S4: Time- treatment interaction terms (P values)**

| **Variable** | **Neuromuscular disease/ Kyphoscoliosis** | **Bronchiectasis** | **COPD** |
| --- | --- | --- | --- |
| PtCO_2_ | 0.38 | 0.52 | 0.87 |
| Minute ventilation | 0.61 | 0.18 | 0.14 |
| Respiratory rate | 0.85 | 0.09 | 0.27 |
| Tidal volume | 0.89 | 0.64 | 0.55 |
| Alveolar minute ventilation | 0.77 | 0.57 | 0.66 |
| Alveolar volume | 0.99 | 0.45 | 0.86 |
| ETCO_2_ | 0.63 | 0.64 | 0.83 |
| Volume of dead space | 0.73 | 0.83 | 0.35 |
| VD/VT | 0.94 | 0.12 | 0.86 |
| Heart rate | 0.15 | 0.60 | 0.57 |

COPD: Chronic obstructive pulmonary disease, ETCO_2_: End tidal carbon dioxide, PtCO_2_: Transcutaneous partial pressure of carbon dioxide, VD/VT: Dead space to tidal volume ratio.

**Appendix S5. References**

1. Miller MR. *et al.* Standardisation of spirometry. *Eur. Respir. J.* **26,** 319–338 (2005).

2. Rudolf M, Turner JA, Harrison BD, Riordan JF, Saunders KB. Changes in arterial blood gases during and after a period of oxygen breathing in patients with chronic hypercapnic respiratory failure and in patients with asthma. *Clin. Sci.* **57,** 389–396 (1979).

3. Domingo C. *et al.* Optimal clinical time for reliable measurement of transcutaneous CO2 with ear probes: Counterbalancing overshoot and the vasodilatation effect. *Sensors* **10,** 491–500 (2010).

4. Kagawa S. *et al.* Initial transcutaneous PCO2 overshoot with ear probe at 42°C. *J. Clin. Monit. Comput.* **18,** 343–345 (2004).
